# Fevers: Their Diagnosis, Pathology, and Treatment

**Published:** 1846-08

**Authors:** 


### REVIEWS.

---

ART. V.—*Fevers: their Diagnosis, Pathology, and Treatment. Prepared and edited, with large additions, from the Essays on Fever in Tweedie's Library of Practical Medicine.* By MEREDITH CLYMER, M.D., Professor of the Principles and Practice of Medicine in Franklin Medical College of Philadelphia; Consulting Physician to the Philadelphia Hospital; Fellow of the College of Physicians, etc., etc. Philadelphia: Lea & Blanchard. 8vo. pp. 604. 1846.

Many have been the dissertations on Fever; long and painfully has the subject engaged the attention of men; and yet it is not exhausted. Fresh investigations are made every year, and new papers and new books, if not new opinions and a new practice, given to the world every season. The journals of this country abound in essays on fever, and histories of epidemic fevers; old works on fever are republished; prizes are offered for the best treatises on this wide wasting disease—all of which illustrates forcibly not only the deep and abiding interest of the subject, but the imperfection of our knowledge in relation to it. Undeniably, the whole grave matter rests still under deep obscurity, after all the labor that has been bestowed upon it. We are not yet agreed concerning the febrile poison, much less respecting the mode by which fever is most safely and successfully treated. The highest authorities are still disputing whether the cause of it be a specific miasm, or mere aerial changes, and the common influences of fatigue, hunger, and exhaustion. The views concerning its cure are not more accordant. Every variety of treatment has its advocates.

The work before us will not settle any of the great questions in dispute, but it is nevertheless an opportune contribution to our accessible literature on the subject. Tweedie's Library is known, and is in the hands of many of our physicians, but Dr. Clymer by revising the articles on fever, and adding the results of his own observation and experience has adapted them more perfectly to the wants of the American profession. In so far as he has engrafted American experience upon the original essays, he has performed a valuable and an acceptable office; but in this particular he has not done as much as might have been expected—certainly not as much as could have been desired. *He* has not exhausted the subject. Still there is wanting a fuller, more elaborate treatise on fevers as they prevail in the United States. What we need is a work descriptive of the peculiar features of our febrile complaints, as seen both in the symptoms and the post-mortem dissections, and of the remedies applicable to the peculiarities impressed upon them by climate and place. But the materials for such a work are hardly yet collected. Countless as have been our histories of particular fevers, very few facts have been published bearing upon their pathology. In other words, we have, up to this time, very few dissections recorded. The histories, moreover, are wanting in minuteness; they deal too much in general description, and give too few details. We have disquisitions in abundance on the "cause of fever," but not many cases accurately described from their origin to their close, with the symptoms each day, the treatment, and the appearances revealed by the examination of the body after death. The execution of such a work, therefore, requires time, but the materials for it are accumulating from year to year, and its appearance may be looked for at no distant day.

The topics embraced in the volume under consideration are the following. General doctrines of fever; Continued fever; Typhoid fever; Plague; Yellow fever; Intermittent fever; Remittent fever; Infantile gastric remittent fever; Hectic fever; Small-pox; Measles; Scarlet fever; Puerperal fevers. We shall confine our examination to a few of these topics, and shall restrict our notice particularly to the contributions of the American editor.

Intermittent fever occupies much space in the volume, and is presented under all its varying aspects. The circumstance which imparts to intermittent fever its highest interest is this protean character—its assuming, now and then, a form of the most extreme malignity, while its usual type is so mild that thousands, in this country, annually suffer it to run on for months, without taking any special pains to check its course. Congestive fever is now well understood to be, generally, a pernicious intermittent. We believe the profession in the United States is indebted to Dr. Bell for, if not the first recognition of this important truth, at least its exhibition in a more striking and convincing light. Since the appearance of his Lectures in 1840 we venture to affirm, the subject of congestive fever has been better understood in the region of the United States where it is endemic, and its treatment, therefore, conducted upon more rational principles. Dr. Clymer has added some important quotations on the subject of these fevers. The following picture will be recognised by many of our readers as a faithful description of a disease with which they are familiar.

"The algid form (with prolonged and icy coldness), of pernicious intermittents is very peculiar. Dr. Maillot contends that it is not an indefinite prolongation of the cold stage. He says in the first stage of an intermittent, the sense of cold experienced by the patient is out of all proportion to the actual reduction of temperature; whereas, in the algid fever, although the skin is icy-cold, the patient does not complain of coldness. And this cold state supervenes after reaction has commenced, and often suddenly. The circulation becomes disturbed, lowered, and the pulse can scarcely be felt, the temperature of the body at the same time rapidly decreasing. The extremities, the face, the trunk become cold in succession, the abdomen remaining longer warm. The skin has the coldness of marble. The tongue becomes pale, moist, and cold; the lips are without color, and the breath is cold. There is no thirst, and attempts to drink often excite vomiting. The actions of the heart become feeble, and only appreciable by auscultation. The intellectual faculties are undisturbed, and there is a sense of repose which is agreeable to the patient. All facial expression is lost. With this state, cholera may become conjoined, and the eyes then become hollow, glassy, and surrounded by a bluish circle. The approach of the algid form is so insidious as often to be mistaken for a remission produced by bloodletting, and the practitioner is only undeceived by the suddenness of the death of the patient. This deceitful calm is very strongly pointed out by M. Bailly, in his chapter on Diagnosis: he says that the patient may be walking about a few instants before the last attack: the accession is sudden; he lies down and dies in a few hours. Even when the pain (of the abdomen) and the danger are both considerable, the face has the appearance of calmness, as if its expression was no longer associated with the sufferings of other parts. Whenever says Dr. Maillot, a sudden retardation of the pulse succeeds to reaction, and there is paleness of the tongue and discoloration of the lips, we should not hesitate to pronounce the case algid. Temporising measures will be followed by death in a few hours. The patient dies as by an arrest of the innervation. If death does not take place, the pulse rises, the skin reacquires its natural warmth, and sometimes yields to remedial measures. The resemblance between this condition and cholera is commented upon by Dr. Maillot."

We quote also what he says respecting the treatment of this form of fever.

"In the congestive form vigorous practice is urgent. Maillot gives an example in which 40 grains of the sulphate of quinine and 2 drachms of ether were given in 4 ounces of water at two doses, in the course of an hour; a starch opiate injection, with 60 grains of the sulphate of quinine and 2 drachms of ether, was ordered at the same time, with sinapisms to the legs and blisters to each thigh. Under this treatment the patient began to recover warmth in a few hours, and the heart to act more forcibly; but the next morning the amendment was so slight, that a sinapism was applied to the whole length of the spinal column, and a clyster with one drachm of sulphate of quinine, and three drachms of ether administered; reaction followed with recovery.—Every effort must be made to produce speedy reaction. Stimulants should be freely given—brandy, ammonia, and particularly capsicum—both by the mouth and rectum; bottles of hot water, and hot bricks are to be applied to the extremities; sinapisms to the trunk and extremities, with turpentine fomentations to the chest and abdomen. As the pulse becomes developed, this active and violent treatment must yield to milder stimulants and diaphoretics. Quinine should be administered freely in large doses, by the stomach and in enemata, and it may also be rapidly introduced into the system through blistered surfaces, produced by the application of ammonia, and by inunction."

The remarks of Maillot will be read with more interest when it is known that, as physician in the French army, in Algeria, his opportunities for observation were most favorable, and that the climate in which he acquired his experience is not unlike that in which our worst cases of congestive fever occur. The reader cannot fail to be struck with the similarity of his practice to that which is now generally pursued by our southern brethren in this form of fever. This coincidence in the type and treatment of a disease prevailing on distant continents but in nearly the same latitude, is striking, while it is what should be expected. The geologist finds the same fossils among the silurian and devonian rocks of Germany and Russia, that we collect on the falls of the Ohio in the same formations. Similarity of geological structure is attended by similarity of animal form and organization. The shells and corals which are found dispersed through the limestone beds of Europe are recognized, even as to the species, in the same deposits in America. So it is concerning disease. The complaints that infest southern Europe bear a strong resemblance to those which prevail on the lower Mississippi; and the fevers of Africa are the congestive fevers of our southern States. Identity of climate and other physical circumstances is accompanied by identity of disease. This part of the treatise under review might have been advantageously extended, and we are somewhat disappointed that, considering the great interest felt in congestive fever, Dr. Clymer did not make larger additions to Dr. Shapter's otherwise very full and complete chapter on intermittent fever. His additions are excellent, so far as they go, but the reader of a work on fever will expect to find more than this contains on one of the most dreaded of all the forms of that disease. This omission evidently does not result from any indisposition on the part of the editor to labor, or from any want of information on the subject. On the contrary his notes evince uncommon industry and extensive research. In subsequent editions the deficiencies of the volume on this head, no doubt, will be supplied.

The disposition shown by ague to return after it has been checked, and assume an obstinate chronic form is the most serious feature in the disease. This tendency, it is conceived, is greater now that quinine is in such universal use than it was formerly when the bark was employed. However this may be, it is well known that although chills are easily arrested by this admirable extract of the bark, they are exceedingly prone to recur, and by persisting for a long time introduce other maladies more formidable than the original disease. In such cases it is believed by many practitioners bark is the most certain remedy; but in our experience nothing has succeeded so well in effecting a radical cure as the preparations of *iron*. These are combined advantageously with some of the bitter tonics, as an infusion of the bark of the wild cherry, or of camomile flowers, or gentian, and by a persevering use of these articles we have rarely failed to see the ague perfectly subdued. The form of iron we generally prescribe is the *precipitated carbonate*, but we are not aware that it possesses advantages over many other preparations. The prussiate we have often given and either of these salts may be administered in the form of pills, of five grains, three times daily. In cases where the spleen has become enlarged, we direct the *iodide* of iron, in pills prepared according the following formula.

"Take of iodide of 127 grains, iron wire about the thickness of a thin quill, half an ounce, distilled water 75 minims. Agitate them briskly together in a strong ounce phial, provided with a well-fitted glass stopper, until the froth which forms becomes white, which will happen in less than ten minutes. Pour the liquid upon two drachms of finely-powdered loaf-sugar in a little mortar, and triturate immediately and briskly for a few minutes; add gradually a mixture of the following powders, viz: liquorice powder half an ounce, powder of gum arabic a drachm and a half, and flour one drachm. Divide the mass into 144 pills."

One of these to be taken before each meal. Purgatives are necessary in conjunction with the alterative tonic. This condition of the spleen, if not obviated, is pretty certain to give rise to dropsy or other grave disorders.

Dr. Clymer has incorporated with the chapter on Remittent Fever the observations of a number of American physicians, which add essentially to its interest. This fever, as it is the most prevalent amongst us, after the intermittent form, and the most fatal, has long attracted a larger share of the attention of southern physicians than any other disease. Like intermittent fever, it often puts on the congestive type, and is then one of the most fearful of diseases. We have observed that men between the ages of fifty and sixty are particularly liable to attacks of this fever, and that in such cases it is particularly fatal. The disease in them is decidedly remittent, and almost intermittent, for several days, when, after a chill, symptoms of fatal congestion ensue, and the case becomes hopeless. We give entire what Dr. Clymer adds on the treatment of this fever:

"In the treatment of remittent fever in this country, the simple expectant plan is the one which has been generally of late recommended by those who have had much experience in the disorder. General blood-letting is in most cases not required, particularly in our southern latitudes, where great caution is required in its use; it is, however, said to be better borne by northerners in such cases, than by the native inhabitants. When decided indications exist for the use of the lancet,—general arterial excitement, as shown in the wild bright eye, intense cephalalgia, hard, full pulse, hot surface, and injected face—it should promptly be resorted to, and great relief will frequently follow; but it will rarely be found necessary to repeat it. Local bleeding, on the contrary, is most generally of great utillity, in relieving many of the prominent symptoms,—as the gastric irritability, tenderness or pain of the epigastric and hypochondriac regions, and the pains in the back. Emetics are now generally abandoned in this form of fever, it being found that they aggravate the gastric distress, which is usually so annoying. If, however, it is found necessary to empty the stomach at the commencement of an attack, the mildest means that will effect the object, should be resorted to. A full dose of calomel (grs. x.) may be given in the first paroxysm, followed by castor oil, or a saline cathartic. No benefit can be derived from a course of systematic purging afterwards, and much harm may result. It is important, however, to keep the bowels open with mild laxatives, care being taken to select those which may not offend the stomach, and by enemata. As to the specific influence in this disease of mercury, no satisfactory evidence has, in the opinion of the writer, been adduced of its utility. Small doses of calomel, blue pill, or hydrargyrum cum creta, combined or alone, may be highly useful as adjuvants in the general plan of treatment, as in that of other diseases. The apartment of the patient should be kept cool, and he should not be oppressed by too much covering to his bed. Cold sponging, with water, or vinegar and water, is generally excessively grateful, when the skin is hot and dry. Dr. Dickson speaks highly of the effects of cold water, and particularly of the cold affusion, which he is disposed to regard as among the most efficient of our febrifuge remedies. 'All that we can hope or anticipate from blood-letting,' says he, 'may be obtained in a majority of cases by the use of the bath, while the latter possesses this striking and obvious advantage, that we can repeat it as often as the symptoms are renewed that require it. Nor can I help expressing my surprise at the very limited resort of my professional brethren to it, when I consider how instinctively we desire it as a relief from the burning heat that oppresses us, and how certain and immediate a means it is of affording this relief. Of the three modes of employing it, namely affusion, immersion and ablution, the first is the most impressive and efficacious, the last the least liable to objection or risk in doubtful cases. The particular indications which demand the resort to it, unhesitatingly, are found in the youth and general vigor of the patient, and the heat and dryness of the surface. The local determination which it controls most promptly is that to the brain, shown by headache, flushed face, red eyes, delirium, etc., with a full, hard, bounding pulse. Seat your patient in a convenient receptacle, and pour over his head and naked body from some elevation, a large stream of cold water; continue this until he is pale, or his pulse loses its fullness, or his skin becomes corrugated, and he shivers. On being dried and replaced in bed, a genial sense of comfort and refreshment will attest the benefits derived from the process, which, as I said above, may be repeated whenever the symptoms are renewed, which it is so well adapted to remove. If the shock of this shower bath or cataract be too great, immersion, which many prefer, may be substituted. Few shrink from this, and almost every one will evince the high gratification and enjoyment derived from it. One of the pleasantest effects following the bath, is the complete relaxation of the surface which it so often brings on, attended with a copious and salutary sweat. I need not warn you against the nearly obsolete practice of endeavoring to accelerate or increase this by wrapping in blankets, or shutting up the apartment, or warming it artificially. The patient is to be covered agreeably to his sense of comfort; and though I would not place him in a current or draught of air, I would have his chamber fully and freely ventilated, Some have strangely enough imagined it to be necessary that evacuations of some kind should be premised to the application of the cold bath, but this is a worse than superfluous caution. It does positively harm by postponing the remedy until the time of its most special adaptation and great utility is past—the earliest and forming stage of the febrile attack. It is here, I repeat, that you will find it most admirably beneficial. Yet you will meet with frequent occasion to advise its repetition at intervals, throughout the whole progress of the disease; and even when the patient can no longer bear either affusion or immersion, he will often be relieved and gratified, by washing and sponging him, especially over the hands, arms, breast, feet, and legs. In the very latest stages of our worst fevers, ablution in this way with ardent spirits, is found singularly refreshing. The affusion of cold water locally upon the head in a stream of some height by the spout bath, is of inestimable advantage in cases where the cerebral determination is inordinately violent, dangerous or tenacious; and will bear to be repeated far oftener than it would be proper to take the patient out of bed for the administration of the general bath. Support him in a leaning posture over the bedside and dash the current from a pitcher over the vertex for some minutes and from some elevation above him. Many who dislike all the other modes of using cold water, entreat for this operation as the most soothing of possible indulgences; nor have I yet met with any ill consequences from allowing its most unlimited frequency of repetition. The cold bath in its several modes of general application is prohibited, let me remind you, when the patient is of feeble habit of body; much advanced in age; much exhausted or enfeebled at the time; when the pulse is weak or the skin cool, or covered with moisture; when the lungs are oppressed or inflamed; and when diarrhœa is present. Its repetition is forbidden when it has occasioned a protracted chill or rigor, or the patient has continued to feel cold or uncomfortable from it.' Excessive irritability of stomach is a frequent and very troublesome symptom. Ice in small pieces, the effervescing mixture, and other remedies of this kind may be administered for its relief. To moderate the excessive thirst, ice, iced water, and acidulated drinks may be allowed. Lemonade, tamarind water, and a weak solution of cream of tartar, are, in general, very grateful. With regard to the administration of quinine some difference of opinion prevails. As a general rule, the more decided the remission, the greater its utility; in the inflammatory form, it is generally of little advantage. Where the remissions are well marked, or where there is a tendenccy to prostration, it is, on the contrary, of the highest value, and should be administered in doses of from five to ten grains, frequently repeated before the anticipated exacerbation. Stimulants sometimes are necessary towards the termination of the disease. Of these, capsicum, wine, brandy, and ammonia, with infusion of serpentaria or valerian, and nourishing food, are very beneficial. Convalescence should be carefully watched, as relapses are easily induced by imprudence. The diet should be digestible and nutritive, and the body should be well guarded against any vicissitudes of climate. The secretions generally should be attended to, and moderate exercise prescribed with the returning strength. The mild bitters will often materially assist us in restoring the powers of the stomach.

"The congestive form of remittent fever requires prompt and vigorous treatment. The chief indications are to procure reaction, and to prevent a recurrence of the paroxysm. To effect the first, sinapisms should be applied over the stomach, chest, between the shoulders, and on the extremities. Blisters are recommended by some practitioners as preferable, whilst others advise that they should replace the sinapisms, when these have commenced to excite irritation of the surface. Stimulants must be freely given internally, quinine, camphor, capsicum, &c. The quinine must be given in large doses, (grs. v. to x.,) at short intervals; it should be combined with capsicum or camphor, or if there is severe vomiting, the oil of turpentine may be substituted for the camphor. The evidence in favor of large doses of quinine repeatedly given in congestive fever, is ample and convincing. Calomel is generally combined with the above remedies, and is administered during the paroxysm, to the amount of fifteen or twenty grains."

This is an admirable summary of American practice in bilious remittent fever, and we commend it to all who have not yet adopted a system satisfactory to them. Much stress is laid upon the use of cold water, but not too much; it would be difficult to exaggerate the virtues of cold water as a febrifuge. Nor is ice, as a remedy in cases of gastric irritability, too highly extolled. We have heard much of medicines "acting like a charm," but if ever we saw a magical effect from any remedy, it has been from ice in fevers, among children and adults, attended by obstinate vomiting. We attach great importance to quinine in remittent fever tending to an intermittent type. If there be a distinct cold stage this medicine will generally be found admissible, and its efficacy is hardly less paramount then than it is in intermittent fever. Every case of remittent fever ought to be narrowly watched, and if it be discovered that at a particular period in the day the patient's extremities grow cold, and other indications of a chill appear, quinine should administered at hours and in doses to anticipate the next paroxysm. We have a painful conviction that we have seen valuable lives lost under a different practice, which might have been saved by such a procedure as this. Their fevers have gone on under the purgative and emetic treatment for nearly a week, exhibiting distinct remissions daily, or every other day, until the true congestive character has been assumed, and all means become unavailing. What might not quinine have effected under such circumstances? Quinine, no doubt, has aggravated the symptoms in many cases, for it is not a medicine that can be used without discrimination; but whenever, and as soon as it can be given without adding to the febrile excitement, it is a remedy more efficacious than all others in fevers. It is a true *febrifuge*, and the true practice is to administer it the first hour that it is admissible.

We shall not attempt to give any farther account of this volume. These points we have noticed in advance of its circulation, as they are matters of special interest to the readers of our Journal at this season. The work will have, as it deserves, a wide circulation, and no one who becomes its possessor will regret the time and money that he may expend upon it. Some of the best minds in the profession have been engaged in its preparation. Christison, Shapter, Burrows, Gregory, Locock, are names familiar to all the readers of modern medicine. To their essays, all the more finished and spirited because the authors had choice of their subjects, the American editor has added a chapter on typhoid fever, besides appending valuable remarks to all the subjects on which the labors of his countrymen have reflected any light.

