# Errata

**Published:** 1846-09

**Authors:** 


ERRATA.—In our last number, at page 141, in the formula for iodide of iron pills, "*iodide of*" occurs where it should be iodine. In the present number Mr. Walshe's name is incorrectly printed "Walsh."

